# Supplementary figures and images for: Evaluation and Genetic Analysis of Parthenocarpic Germplasms in Cucumber
Source: Genes (Basel). 2022 Jan 25;13(2):225. doi: 10.3390/genes13020225 (PMC8872377; doi:10.3390/genes13020225)

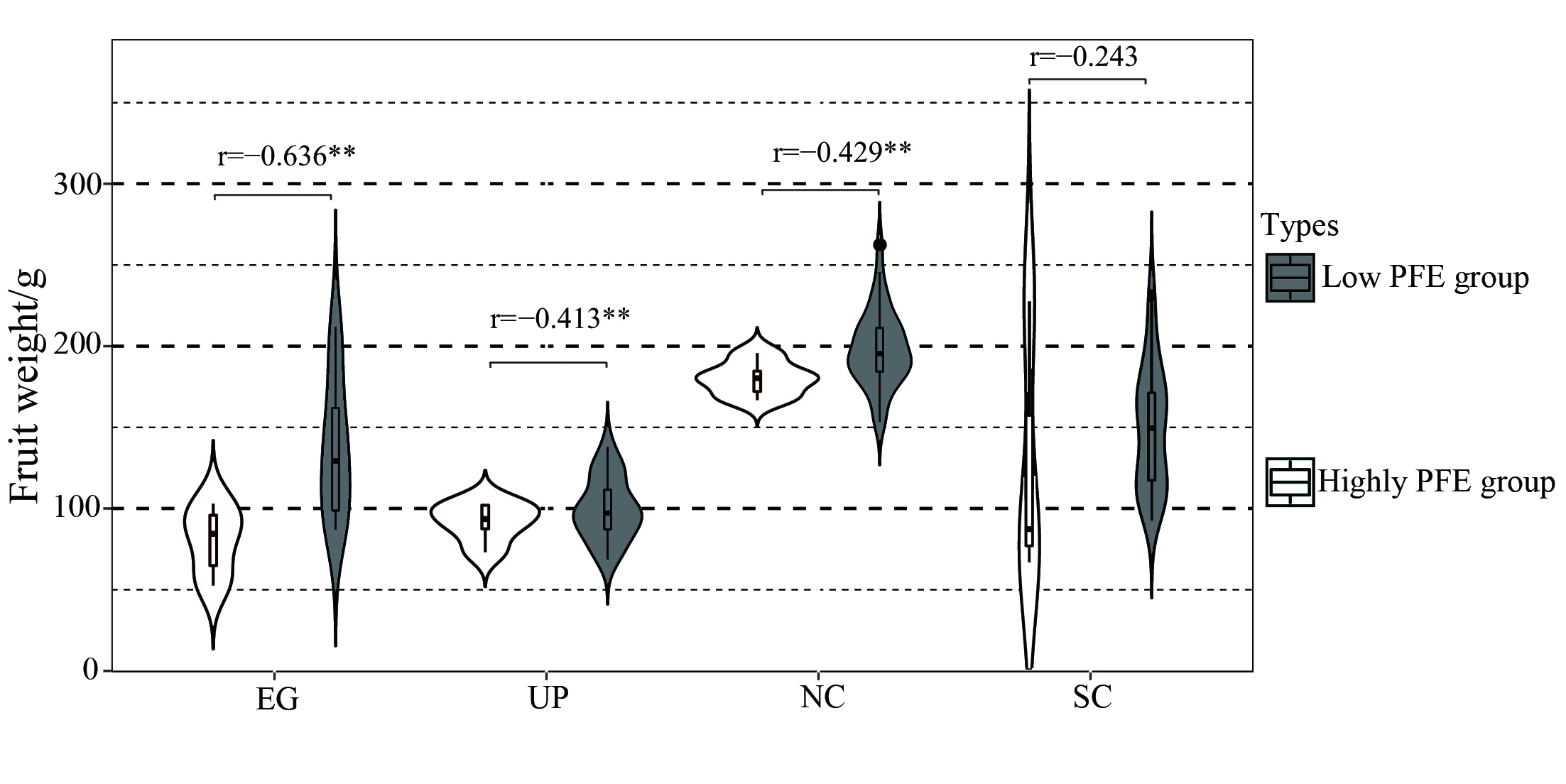

Supplement: Supplementary file 1 [file genes-13-00225-s001.zip › Supplementary Figure S1.jpeg]
